# Evolution and Biogeography, and the Systems Measurement of Mammalian Biotas

**DOI:** 10.3390/life13040873

**Published:** 2023-03-24

**Authors:** Charles H. Smith, Patrick Georges, Ngoc Nguyen

**Affiliations:** 1 Western Kentucky University (Prof. Emeritus), Bowling Green, KY 42101, USA; 2 Graduate School of Public and International Affairs, University of Ottawa, Ottawa, ON K1N 6N5, Canada; pgeorges@uottawa.ca; 3 Department of Mathematics, Western Kentucky University, Bowling Green, KY 42101, USA; ngoc.nguyen@wku.edu

**Keywords:** mammals, faunal regions, classification, natural systems, evolution, Spinoza

## Abstract

Biological evolution is generally regarded as a stochastic or probabilistic process, per the ideas of Darwin in the nineteenth century. Even if this is true at the meso-scale, it still may, however, be impacted by overarching constraints that we have not yet identified. In this paper, we revisit the subject of mammal faunal regions with a mind to explore a potential kind of macroevolutionary influence. We first identify an optimum seven-region mammal faunal classification system based on spatial and phylogenetic data from a comprehensive 2013 review, and then examine the possibility that this classification provides supporting evidence for a Spinoza-influenced philosophical/theoretical model of the “natural system” concept developed by one of the authors in the 1980s. The hierarchical pattern of regional affinities revealed does do this.

## 1. Introduction

In 1983, CHS published a trio of papers [1,2,3] outlining an arrangement of family level-based mammal faunal regions. The first two of these papers featured two main innovations: the application of geographic entropy maximization methods [4] to identify distance-decay characteristics of faunal relations among the regions identified, and an argument supporting a more enlightened means of assessing relative regional equivalencies. The third paper applied second-order statistics (largely, statistical moments) derived from the first two to produce world maps conveying representations of the way mammalian faunas are interrelated; for example, illustrating spatially varying mean and total cosmopolitanism characteristics.

The four-region, ten-subregion system identified in [1] included a new subregion covering Mediterranean and West Asian lands, termed the “Tethyan”, that was subsequently used by later researchers to help characterize certain faunal patterns, for example of bats [5,6], amphibians [7], ticks [8], rodents [9], and snakes [10]. The methodology itself, however, has not seen much development, although Wilson [11] (p. 868) specifically cited the work in his study of Boltzmann-type statistical physics models as applied to complex systems, noting: “using the Boltzmann methods…this gives a new ecological model. There is only one empirical example in the literature known to the author [1]. There are two elements of knowledge transfer from urban to ecological modelling here: first, an explicit and potentially more effective way of handling spatial interaction and, second, the ways of analysing the dynamics of spatial patterns”. Whittaker et al. [12] (p. 2210), in introducing a special issue on regionalization systems, commented

“Smith undertook a pioneering analysis designed to assess the efficiency of this system and whether it provided a logical set of relationships among the subregions, i.e., how well they nest within the higher level regions. Smith [2] wrote an innovative companion paper which set out to assess the proposition that evolution might be regarded as a stochastic spatial process with inter-regional affinities explicable through chance interactions between the subregions and a deterministic distance-decay effect on the diffusion of evolutionary innovations. This approach was in some ways rather ahead of its time, as the computer power and computerized distributional datasets required to develop this line of thinking further were lacking in the early 1980s.”.

The present study, in some ways, parallels Smith’s 1983 analyses, but draws on new data presented by Holt et al. [13] to characterize terrestrial vertebrate faunas; that is, it employs a more recent biological and regional mammal classification system methodology, making use of a more current conception of phylogenetic origins. Here, we identify a set of mammalian faunal regions that most parsimoniously summarizes that more modern data set, but, beyond this, the results are examined as a possible improvement over the 1983 results, both in terms of their efficiency, and their possible relation to a “natural systems”-related theoretical model.

## 2. How Many Regions?

Holt et al.’s 2013 study [13] left unanswered the question of what number of regions might most efficiently represent the biogeographic regionalization patterns in the Mammalia. The authors likely avoided the issue because of “hierarchy bias”; that is, a sense that a tree-organized arrangement of inclusiveness not strictly mirroring cladistic systematics is not conducive to a “natural” geographical grouping of forms (i.e., because such cannot exist, even in principle). Beyond the cladism assumption, this bias also stems from more than a century’s worth of discussion as to whether observed faunal regions (describing whatever living forms) are “equivalent”: that is, the fact that any such arrangement will include some regions that host more forms than others, and/or have higher or lower numbers of endemic taxa. Among the most common of these criticisms has targeted the relative similarities and low endemism rates of the Wallacean/Sclaterian Palaearctic and Nearctic regions as compared to, say, the same scheme’s African and South American regions. In 1894, Wallace opined: “This question of equality is decided almost exclusively by one characteristic, and one that seems to me to be not the most important for the purpose we have in view. This character is the possession of peculiar groups of the rank of family or order, taking no account either of the richness and variety of life-development, or of the geographical extent of the area in question” [14] (p. 613). However, and beyond Wallace’s point, should we even assume that in a probabilistically-evolving system the diversification principles involved must lead to “faunally equivalent” components?

A primary objective of Smith’s 1983 papers [1,2,3] was to expose this “hierarchy bias” by showing how a particularly efficient n-region classification: (1) might be expressed in ordinal terms (via multidimensional scaling and other methods) as a maximally diverse grouping, and (2) could involve regional structures whose individual diversity characteristics were equally well specified through a combination of entropy maximization and multiple regression operations based on system-level, rather than individual regions-based, statistics. The ten-region classification in fact produced very high levels of internal specificity, independent of levels of endemism or taxon richness in its individual units. The implication is that a macroevolution process taking place on a limited planetary surface produces long-term results which, though consistent with a historical process of species lines divergences, nevertheless emerges probabilistically in response to very complex long-term interactions among biological/environmental/geologic constraints and opportunities. This implies that the distance-decay variations identified in the papers result not only from simple varying linear distances, but from a myriad of intervening, and recurring, historical/environmental factors as well.

Here, the Holt et al. [13] spatial divisions (as set out in its Figure S4C) were used to assemble a group of five faunal regions models, the most inclusive involving a five-region system, and the least inclusive involving nine (see Tables ‘Correspondence Table’ and ‘Data 1’ in the Supplementary Materials). Smith’s 1983 inclusion methodology was used to assign those families present to individual regions, leading to the construction of five similarities matrices (i.e., of 5 × 5, 6 × 6, 7 × 7, 8 × 8, and 9 × 9 elements) relaying inter-regional mammalian affinities. The question asked was could any one of these produce an arguably single most superior regional classification?

Before presenting the results of this analysis, consider again the Holt et al. data itself. Seemingly, one good way of assessing the “most efficient” clustering level would be to examine the co-phenetic values attached to each level of the hierarchy, one after another, looking for statistical gaps in the progression indicative of break points in the relation between variation explained and degrees of freedom. The overall co-phenetic score of 0.77 in the Figure S4C dendrogram identifies the first regional split, between Australia/New Zealand and all other areas. The scores from that point on, at each further split, are in order: 0.67, 0.61, 0.58, 0.54, 0.45, 0.44, 0.42, 0.42, 0.37. 0.37, 0.36, 0.36, 0.34, and so on. At once, a significant gap seems evident, between 0.54 and 0.45; this gap becomes more apparent when one converts the scores to the progression of improvements in score (i.e., the percentage increase from item to item), the sequence now becoming 0.230, 0.122, 0.090, 0.052, 0.078, 0.162, 0.031, 0.033, 0.005, 0.109, 0.004, 0.028, 0.009, 0.038, etc. The 0.162 figure stands out as the most extreme downward trend reversal, identifying a six-region model consisting of the units: Australia, South America, Holarctic, Africa, Southeast Asia, and Madagascar. This is not so surprising, as apart from a single Holarctic unit and the addition of Madagascar, this is quite similar to the old Sclater–Wallace model (the second-best figure, 0.109, identifies a ten-region model, fairly consistent with Smith’s 1983 results).

However, there is a problem. The Holt et al. effort, treating terrestrial patterns only, ignores marine mammals, which we cannot do here if we intend to examine the entire pattern of probabilistic mammalian biogeographic radiation/evolution. Since a regional unit of marine-areas life would surely represent an early-order addition to the Holt et al. dendrogram, this suggests the best working hypothesis for a most efficient classification might be a seven-region model. In our test classifications into 5, 6, 7, 8, and 9 units, therefore, we expected that summary statistics attached to the seven-region model would reveal this as well.

Before setting out the descriptive statistics, some nomenclatural clarifications need to be introduced. Therefore, let us assume five different models where the world can be rearranged or decomposed into either 5, 6, 7, 8, or 9 variably-sized geographical regions, with the total number of regions included in each model being a variable noted *r*, with *r* = 5,…9. Each geographical region in any such model will be referred to with an index *i* = 1, 2,… to *r*. Mammal families are indexed as *f* = 1 to *N* where *N* is the total number of mammal families in the world (140, after [15]), as applied here. Now, let us denote

*d_f,i,r_* for *f* = 1,… *N*, *i* = 1,…, *r*, *r* = 5,…, 9; a dummy variable that takes a value of 1 if there is such a family f in region *i* of the *r*-region model, and 0 otherwise. The collection of *d_f,i,r_* forms a Table of presence (1) and absence (0) of mammal families across all regions *i* for any model *r* (see Sheet 3 of the Supplementary Materials).

*div_i,r_*, *i* = 1,…, *r*, *r* = 5,…, 9; the diversity of (i.e., the number of families found in) each region *i*, in each model with total number of regions *r*, with $div_{i,r}=\sum_{f=1}^{N}d_{f,i,r}$(see Sheet 3, Supplementary Materials).

*c_i,j,r_*, *i* and *j* = 1,…, *r*, *r* = 5,…, 9; the number of common families between two regions *i* and *j* in a world of *r* regions (see Sheet 3, Supplementary Materials).

*dis_i,j,r_*, *i* and *j* = 1,…, *r*, *r* = 5,…, 9; the number of distinct families between two regions *i* and *j* in a world of *r* regions. In particular, *dis_i,j,r_*, is the number of forms found in *i* but not in *j*, while *dis_j,i,r_* is the number of forms found in *j* but not in *i* (see Sheet 4, Supplementary Materials).

*s_i,j,r_*, *i* and *j* = 1,…, *r*, *r* = 5,…, 9; the Smith similarity index between two regions, *i* and *j*, in a world of *r* regions with *s_i,j,r_ = c_i,j,r_* − (*dis_i,j,r_ + dis_j,i,r_*) (see Sheet 4, Supplementary Materials).

*z_i,r_*, *i* = 1,…, *r*, *r* = 5,…, 9; the “zeroth moment” (or “total cosmopolitanism”) of a given region *i*. For example, $z_{i=1,r}=\sum_{f=1}^{N}\left(d_{f,i=1,r}+\sum_{i\neq 1}^{r}d_{f,i,r}\right)\;\mathrm{when}\;d_{f,i=1,r}=1\;\mathrm{and}\;0\;\mathrm{otherwise}$ (see Sheet 6, Supplementary Materials).

*z_i,j,r_*, *i,j* = 1,…, *r*, *r* = 5,…, 9, the “zeroth moment” (or “total cosmopolitanism”) of a pair of region *i,j*. For example, $z_{i=1,j=2,r}=\sum_{f=1}^{N}\left(d_{f,i=1,r}+d_{f,i=2,r}+\sum_{i\neq 1,2}^{r}d_{f,i,r}\right)\;\mathrm{when}\;d_{f,i=1,r}\mathrm{and}\;d_{f,i=1,r}=1\;\mathrm{and}\;0\;\mathrm{otherwise}$ (see Sheet 6, Supplementary Materials).

*nz_i,r_*, *i* = 1,…, *r*, *r* = 5,…, 9; the “negative zeroth moment,” the complementary value to the zeroth moment and defined as $\left(div_{i,r}\times r\right)-z_{i,r}$ for diagonal elements (a region *i*), or more generally, for any two regions *i* and *j*, $nz_{i,j,r}=(c_{i,j,r}\times r)-z_{i,j,r}$, which collapses to $nz_{i,i,r}=(c_{i,i,r}\times r)-z_{i,i,r}=(div_{i,r}\times r)-z_{i,r}$ when *i = j* (see Sheet 6, Supplementary Materials).

*z_i,r_/div_i,r_* = the “mean cosmopolitanism” for a region *i* (see Sheet 6, Supplementary Materials).

The similarities scores *s_i,j,r_* defined above and given in Table 1 for the seven-region model are what have been termed “Smith coefficients” [16]; these were used originally in [1]. It is a nonproportional index of similarity producing “intersection set”-like scores rather than proportions, and was used in the 1983 papers [1,2] because the multidimensional scaling methods applied there call for numbers based on observed quantities rather than proportional transformations. The scores it produces are often negative numbers, but one of its useful qualities is that a score of zero signifies a pairing of regions whose faunas contain as many taxa held in common as they have taxa that are not held in common. It has since been used successfully in a music history context to address a similarity of the style of composers (to one another) through the study of common and distinct sets of composer-on-composer influences [17].

As shown above, a good many descriptive summary statistics can be calculated using the statistical moments of the distributions of values involved. Some of these statistics were also calculated in Smith’s three 1983 papers [1,2,3]. For example, a “mean cosmopolitanism” statistic was produced for each region by taking the “zeroth moment” of each and dividing it through by the number of forms present (*z_i,r_/div_i,r_*). So, and for example, in the seven-region classification here, 47 mammalian families are assigned to its Holarctic region component, and these particular 47 taxa appear, in sum, 142 times in the regional tallies worldwide. Overall, 142/47 = 3.021; thus, on average, the families associated with the Holarctic region are found in 3.021 regions in the classification overall. The value 3.021 represents the first moment (mean) of the distribution, whereas 142, the “zeroth moment”, is its “total cosmopolitanism”. Further moments of a given distribution can also be calculated.

All statistics are computed in Sheet 7 and Table S1 of the Supplementary Materials a seven-region world. No. 1: The mean number of mammal families present in the regions in that classification $\sum_{i}div_{i,r}/r$; No. 2: The mean zeroth moment of the regions in that classification $\sum_{i}z_{i,r}/r$; No. 3. The ratio of No. 2 to its corresponding negative zeroth moment $\left(\sum_{i}z_{i,r}/r)/(\sum_{i}nz_{i,r}/r\right)$; No. 4. For each model, the mean of the mean number of regions each subject family is found in overall $\sum_{i}\left(z_{i,r}/div_{i,r}\right)/r$. (For example, for the 5-region model, if there were a particular region with 50 families, and on the average these 50 were found in 2.5 regions in the classification, this would create a zeroth moment for that region of 50 × 2.5 = 125, and that value 2.5 would have to be averaged with the parallel values for the other 4 regions to come up with a 5-region figure—which, in the real data above, is in column 4 and is 2.230); No. 5. The ratio of the sum of the diversities of the n regions to 140, and the overall number of mammal families in the taxonomic classification $(\sum_{i}div_{i,r})/N$; No. 6. The mean of the column means of the n × n Smith scores in that classification, $\sum_{i}((\sum_{j}s_{i,j,r}/r))/r$ (for the 7-region model, that is the mean of the 49 top left values in Table 1); No. 7. The parallel mean of the “forms common to” used to produce the Smith scores,$\;\sum_{i}((\sum_{j}c_{i,j,r}/r))/r$. (For the 7-region model, the mean of 49 top right values in Table 1); No. 8. The mean zeroth moment ‘common-tos’,$\;\sum_{i}((\sum_{j}z_{i,j,r}/r))/r$. (For the 7-region model, the mean of the 49 bottom left values in Table 1); No. 9. The average number of ‘common-tos’ among all combinations of the regions: $\sum_{i}\left(\left(\sum_{j\neq i}c_{i,j,r})/(r-1\right)\right)/r$ (computed similarly to No. 7 above, but ignoring *i* = *j* top right values in the n x n matrices); No. 10. Analogous to Nos. 8 and 9, above, but summarizing the off-diagonal zeroth moment ‘common-tos’,$\;\sum_{i}\left(\left(\sum_{j\neq i}z_{i,j,r})/(r-1\right)\right)/r$.

**Figure 1:**
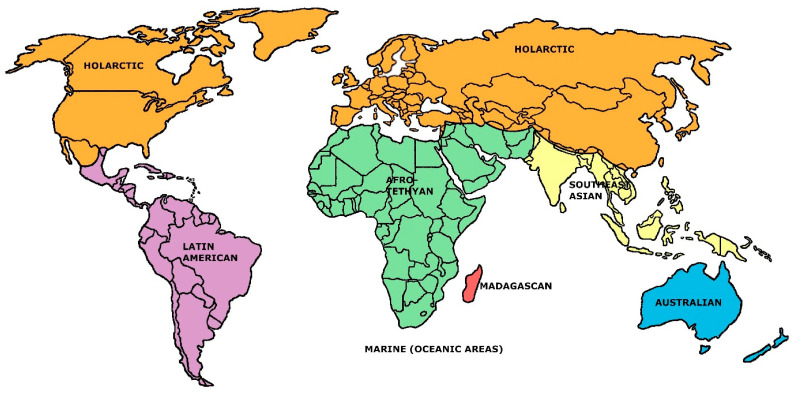
The seven-region mammal families faunal model established here (in part after the data of Holt et al. [13], Figure S4C).

From Table 2, it is apparent that, relatively speaking, the seven-region model stands out as deviating most from the general trends of increase or decrease in each of the descriptive faunal statistics forced by advancing the number of regional units. (The same trend is apparent in some other sets of descriptive statistics that were calculated, but these extend to even more obscure formulations which seem unnecessary to report at this point). The superiority of the seven-region model suggested by Table 2 was further documented by performing linear and log n-linear regressions on each set listed there against the values five through nine: in all twenty analyses, the predicted regression model values for the seven-regions’ cases overestimated the original values (and in both sets of regressions, the sum of overestimations across the ten analyses was greatest for the seven-region models). We believe that this information solidifies a verdict that, from the Holt et al. starting point, a seven-region model stands out as the most efficient general rendering of mammalian biogeographic regions. This rendering is sketched in Figure 1. The associated faunal similarities matrix is given in Table 1.

We now need to demonstrate how efficient this systemization is in specifying itself, as in the 1983 papers [1,2]. A number of analyses were run that paralleled those in the earlier works; in the interest of space, only two of these are profiled here.

**Figure 2:**
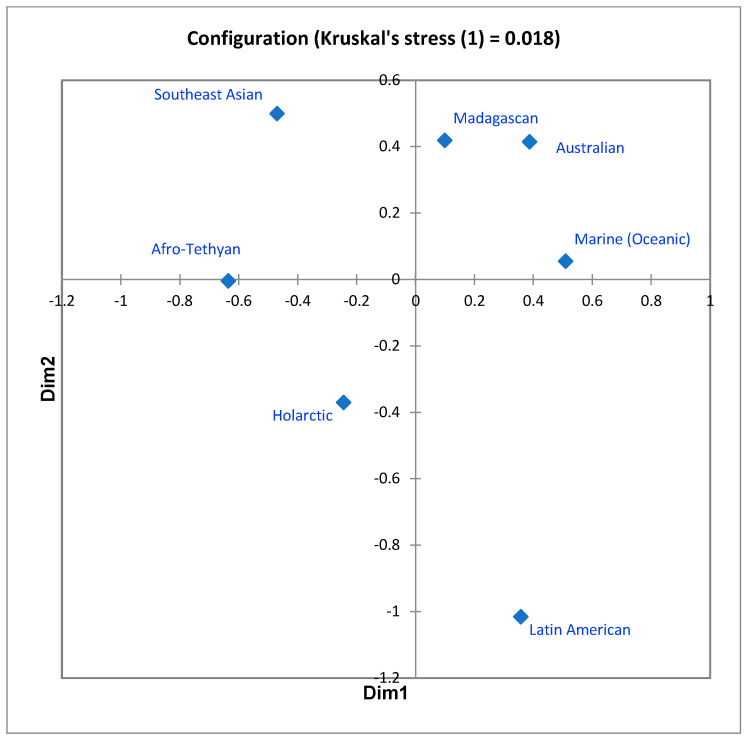
Two-dimensional MDS ordination of the values displayed in Table 1. Stress = 0.018.

In the first, the seven-regions similarities matrix was subjected, as in 1983, to nonmetric multidimensional scaling. The ordination in two dimensions, producing a stress value (Kruskal’s stress (1)) of 0.018, is shown in Figure 2. The units appear quite distinct, though, as anticipated, not equally unique with respect to the system as a whole. More instructive, however, is the way the configuration subtly portrays the difference between “distance decay effect” and literal distance. In the configuration, the Madagascan, Australian, and Marine units are mutual nearest neighbors, while the Australian/Southeast Asian and Madagascan/Afro-Tethyan units, which are physically closer together than other pairings of regions, are further apart. This is counterintuitive, but appears more reasonable when one understands the basis of the distance decay effect to be not only distance per se, but a combination of this with other ecological/historical influences on remoteness and isolation. Australia and Madagascar have each been isolated by water bodies for long periods of time, and while the Marine realm borders all of the other six, residence within it requires such a profound biological specialization that few mammal ancestors were able to accomplish this; in short, ecological remoteness and geological isolation are acting to influence the evolution of mammal diversity patterns as much or more than mere distance.

The second analysis began with an entropy maximization operation; as in the 1983 studies [1,2], this involved double-standardizing the n × n matrix of similarities scores through an operation known as bistochastization. This procedure produces n × n matrices of z scores that iteratively converge on stable final values to maximize the entropy of the original matrix values (see Sheet 7 of Supplementary Materials for these scores and Sheet 8 for R code). (For examples of use, see [2,18,19,20,21,22]). Replacing the original scores with entropy-maximized ones removes the complicating effect brought on by the regions having greatly varying overall diversities. As explained in the 1983 papers, these new scores can be applied to regressions predicting the original values, removing chance effects, with the residuals then being studied for evidence of distance-decay effects among the spatial units. (In the usual regional human geography context, for example involving regional source-destination commuter flows data among a set of towns, the residuals are linked to various measures of actual or implied distances; here, as in the 1983 studies, the descriptive moment statistics are used to the same effect). It was expected that the new data would produce results improving upon those from 1983, and perhaps also tending to add support for the seven-regions model over the others.

In the sets of regressions across the five models, the dependent variables are the vectors of similarities scores connected to each region. For the five-regions classification, there are five regressions, each involving five cases: the dependent variables are the five (different) vectors of Smith index scores, one for each; while the independent variables consist of (1) the parallel five vectors of entropy maximization scores (i.e., the double-standardized z scores), and (2) the mean and standard deviation of the cosmopolitanism values of the faunal families in the different regions of the classification. In [2], Table 3 relays results for the ten-region model discussed there; here, Table 3 below summarizes the results, slightly differently, for the five different regional models.

The first three sets of statistics in Table 3 above convey information on both the relative efficiency of the seven-region classification, and the degree to which the units in the five classifications are equally well specified by the sets of independent variables. Because one of the multiple regressions (in the six-region series) by accident accounted for very nearly one hundred per cent of the variation in that situation, and thus produced an f statistic (8576.3) that was unrepresentatively extreme, the last five sets of means in Table 3 exclude that value: instead, they report different analogous subsets of the remaining values. From these, it can be seen that the seven-region classification in each instance produces the highest mean f statistics. In absolute terms, the mean f statistic for the seven regressions in that group, 531.3, is exactly three times as high as the mean of the analogous ten values depicted in Table 3 of [2].

These data make a further good case for the relative superiority of the seven-regions model. Within the seven multiple regressions described above, there is some considerable variation among them with respect to the significance levels of each of the independent variables, but this is to be expected because, again, the regions are structurally diverse. The simple correlations between the double-standardized scores and their respective dependent variable vectors range in value from *r* = 0.951 to 0.997, but in each case the remaining independent variables combine to account for a very large proportion of the remaining variation (in each model, the overall Pearson’s r^2^ statistic much surpasses 0.99).

## 3. On Evolution

Smith [23] takes the position, after Alfred Russel Wallace, that evolution represents a macro-process served by a variety of known (and very likely unknown) laws of nature. Wallace writes [24] (pp. 3–4) “Evolution, as a general principle, implies that all things in the universe, as we see them, have arisen from other things which preceded them by a process of modification, under the action of those all-pervading but mysterious agencies known to us as ‘natural forces,’ or, more generally, ‘the laws of nature.’” Clearly, Wallace believed that natural selection was one of those “natural forces,” or “laws”: he referred to it as “the law” of natural selection as often as he did, more loosely, as a theory. In fact, his conceptualization of natural selection involved Humboldtian (we would now term “state-space”) thinking; that is, he regarded the phenomenon more as a state of balancing interactions than as a process (as Darwin did). Smith [25] (p. 420) went so far as to proffer: “True ‘Wallacism’, it seems to me, must therefore be conceived as, exactly, ‘natural selection by evolution’”; this emphasizes the notion that evolution produces conditions forcing natural selection-mediated outcomes. The relevance and importance of this to the present discussion is that Wallace’s setting evades a criticism that has always been levelled at the Darwinian model: that it portrays a result—adaptation—that is produced by itself. Some sources have complained that this is tautological thinking (e.g., [26] (pp. 237, 238): “The process is adaptation, and the end result is the state of being adapted. The problem is how species can be at all times both adapting and adapted”), while others (e.g., [27]) have gone to the extreme of urging it is teleological. By contrast, Wallace’s natural selection implies the results of a continuing “elimination of the unfit”; that is, a tendency toward balancing populations with their natural surroundings through an elimination of those individuals that are less successful at continuing to operationalize their niche relations. This reads in modern terms like a negative feedback relation, and to solidify the point in his Ternate essay, Wallace came up with its famous likeness to “the centrifugal governor of the steam engine, which checks and corrects any irregularities almost before they become evident; and in like manner no unbalanced deficiency in the animal kingdom can ever reach any conspicuous magnitude” [28] (p. 62). Bateson [29] (p. 435); [30] (p. 43) even suggested that Wallace might be recognized as the first cybernetician; while this is a bit of a stretch, it is nevertheless true that his position fits nicely into later “push/pull” concepts of ecosystem evolution suggested by Maruyama in his classic “deviation-amplifying mutual causal processes” paper of 1963 [31]. In that work, Maruyama describes how negative feedbacks in the environment might be coupled with positive feedbacks to produce a sustained evolutionary progression. In his 1858 paper, Wallace identifies the three major factors that force an “elimination of the unfit”: namely, variation within populations, organic superfecundity, and limited exploitable environmental resources. To these three, we suggest adding one more: the limited but unbounded surface proper of the earth. Thus, populations are forced to respond to both the limited resources of any given location, and the mixing effect caused by a finite overall space (i.e., populations cannot forever evade place-specific “limited resources” simply by dispersing: they are ultimately forced to cope evolutionarily). Wallace (or Maruyama, for that matter) does not identify suspects for what might qualify as the positive feedback part of the loop, but as Smith has suggested [32,33], this might consist of (1) genetic mutations, and (2) a tendency for populations as a group to disperse nonrandomly—specifically, in the direction of ecological conditions more likely to facilitate generalist evolutionary change over the long term.

These are all theoretical concepts, and it is tempting when turning to the idea of phylogeny to view this as representing, in contrast, a purely factual reality. This is true to the extent that when correctly formulated, a phylogeny accurately reflects the history of the derivations of the groups covered, but at the same time it ignores all those other aspects of planetary processes that fuel such development, and of which it is a part. These processes are myriad in number, operating both organically and inorganically, and at every physical scale. For example, most living cells die and are replaced at rates ranging from days to several years. Further, the molecules making them up turn over even more rapidly, on average. Even small environmental changes can profoundly affect the delivery efficiencies involved, with resulting changes in fitness and likelihood of passing genes on to the next generation. In point of fact, in a state-space sense, we are nearly completely regulated by the exigencies of environmental inputs. It is usually assumed that the management of these interactions, mediated by the memory function inherent in the operation of DNA, proceeds largely in a Darwinian random walk-like manner via natural selection, unfettered by further controlling influences, but this may not quite be the case.

Meanwhile, there has been a tendency to regard regional biotic patterns through balance sheet thinking; that is, with them as the simple net outcome of species divergences, dispersals, and extinctions. While at one level this is difficult to deny, it neglects the fact that organisms do not merely exist within an environment, they more generally speaking are but one element of all manners of turnover *constituting* that environment. The degree to which the historical emergence of new biological forms might have been governed by systemic forces extending beyond simple origination and disappearance phenomena has been but poorly considered. A “geography as handmaiden” approach to evolution tends to set its biological actors apart as separate from the stage, leading to a belief that phylogeny is a direct reflection of the evolution process; while, more likely, it only represents one manifestation of its results.

## 4. Natural Systems?

The classification results presented earlier, though reasonably straightforward, might be criticized as a step backward toward phenetics in a preferred trend toward natural systemization methods. This conclusion seems possible even if our earlier arguments, that origins-oriented vicariance modelling does not fully capture the nature of worldwide evolution processes, are accepted. However, are such surmises necessarily apt to begin with?

Around the same time his 1983 papers appeared in print, CHS was considering alternative ways of framing the ‘natural system’ concept. Although the Darwinist program is based largely on random walk notions of process, perhaps this is an oversimplification, and some kind of probabilistic—though systemic—constraint factor exists that arranges system differentiations into discrete, but inter-organized, subunits. The engineer Adrian Bejan [34] has stated “for a finite system to persist in time. It must evolve freely in such a way that it provides easier access to the imposed currents that flow through it, “ but his approach (the so-called Constructal Law) does not identify actual systemic constraints, independent of system type or scale, that might super-organize such a process. Neither does another well-known system attempt to understand macroevolution, the “nonequilibrium“ theory of Wiley and Brooks [35].

A possible systemically-organized macroevolutionary influence occurred to CHS on his exposure to the writings of Rationalist philosopher Benedictus (*aka* Bento, or Baruch) de Spinoza (1631–1677). Spinoza’s natural philosophy begins with the posed existence of what he termed “Substance”, an exhaustive natural reality whose tangible expression is rendered through “Attributes” (capital “A”) he termed “Thought” and “Spatial Extension”. Unlike our present conception of attributes (small “a”), these are in effect “rules of order” that, independently of one another, frame the organizational basis of the whole of natural structure. More on this is out of place here; for further information, see Nadler [36,37]; Spinoza [38].

**Figure 3:**
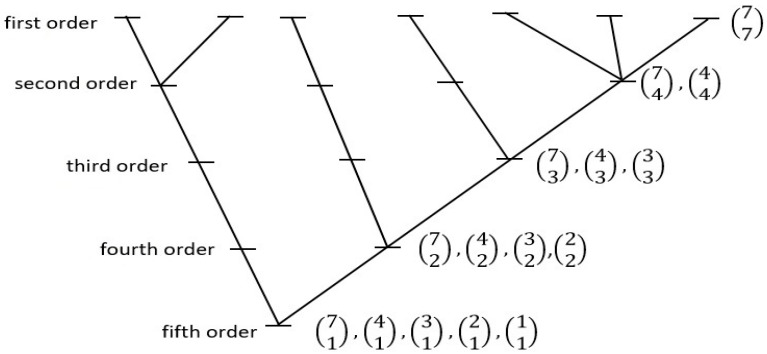
One of the two possible seven-tipped “maximum-likelihood trees” of hierarchical relations constructed on the basis of combinatorial mathematics. Within this structure, all class-subclass sub-hierarchical relations are ordered on a “most-probable-state” basis. Thus, beginning at the $\left(\begin{array}{c} 7\\ 7 \end{array}\right)$ level, the seven first-order subclasses (here, “tips”) combinatorically group most frequently into one second-order class containing three first-order subclasses, one containing two subclasses, and two containing one subclass (and not, for example, into three second-order classes containing two first-order subclasses each, and one containing one first-order subclass). All other combinatorically-defined relations noted observe the same basic “lazy universe” plan. Trees with two, three, four, five, six, or seven tips can be identified that observe this system-wide rule of construction, but there are no eight-, nine-, or ten-tipped arrangements that extend the series under the same conditions. (For further explanation see [33] (pp. 370–375), or the sub-section titled ‘A Model of Hierarchical Internal Relations’ at https://people.wku.edu/charles.smith/once/syst1.htm, accessed on 10 January 2023).

**Figure 4:**
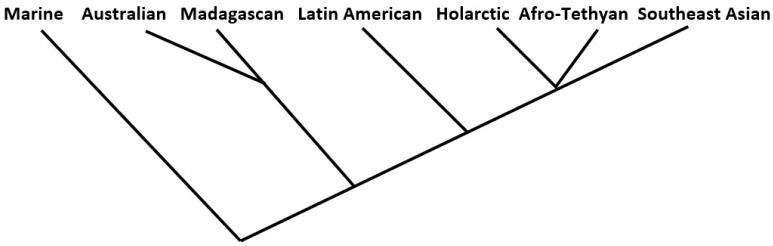
Cluster analysis arrangement of the regions resulting from 10 of the 144 individual analyses performed here.

CHS has argued [21,33,39,40] that Spinoza’s philosophical understanding might also have scientific merit if all natural systems are underlain by subsystemization arrangements that are in line with his concept of “Attribute”. For one possible such arrangement, CHS considered what kind of principle of branching, hierarchical, development/system memory might emerge, in all natural systems, as a simple probabilistic process; that is, as organization worthy of the word, yet actualizing in a “lazy universe” fashion. The model posed—one that might sustain any given natural system—invoked the concept of the “maximum likelihood tree”, a developmental branching organization based on simple considerations of combinatorial likelihoods. In this kind of tree, all hierarchical class-subclass relations are defined strictly on the basis of each such relation having come about as the result of maximum likelihood combinatorial arrangements (that is, as the most frequent way they would naturally sort out on coming into existence). For a seven-tipped system, there are only two equally probable such hierarchical trees; one of these two is shown in Figure 3 (the other will appear in Figure 4). See Figure S1 in the Supplementary Materials for a more general graph from which Figure 3 is excised.

A few years later, CHS developed an interpretation of the other fundamental Spinozian Attribute, Spatial Extension. This was based on the notion that within any natural system, some small number of functional subunits might exist that (1) exhaust the whole of the structure, and (2) interact among themselves, sharing energy and information, in such a fashion as to generate a dynamic balance contributing to the system’s integrity, and, ultimately, to the fact of spatial extension itself. Mathematical simulations were carried out in an effort to determine just how many interacting subsystems might be needed to sustain such structures; it turned out that only systems comprised of four-subsystem interrelationships could [21,39,40]. Several pilot studies have been carried out by CHS on real world systems relatable to this second Attribute, where applicable forms of pattern-related data are available: these include a regional human population system, the internal structure of the earth, topographical patterns within drainage basins, worldwide topographical trends, color patterns on butterfly wings, and amino acids structure (see Smith, http://people.wku.edu/charles.smith/once/writings.htm#2, accessed on 10 January 2023). All of these efforts returned hopeful results, but it was not until the 2010s that more controlled analyses (on two geophysical settings) could be performed, and were published [21,39]. These studies did produce considerable support for the overall model.

The first of the two Attribute models is the more difficult to test empirically; it projects relationships that are cumulative over time, but usually in a manner reflecting complex historical forces (as distinct from singularly-originated phenomena such as phylogenies). Still, the deduced seven-subclass structure is a good clue, and there are natural emergent systems that seem to fit this model. One is the organization of the major structural divisions of Earth, consisting precisely of seven zones: the inner and outer cores, mantle, oceanic crust, and continental crust, hydrosphere and atmosphere. These seven may also rationally be arrayed, in just this order, to label, left to right, the seven tips displayed in the maximum-likelihood tree displayed as Figure 3 above. A predominantly seven-unit organization is also manifested in the present size distribution of tectonic plates: the “List of Tectonic Plates” Wikipedia entry gives a consensus rendering of the sizes (in million km^2^) of the seven largest: 103.3 75.9 67.8 61.3 60.9 58.9 43.6; after these, there is a sudden nearly threefold reduction in areal extent to the next largest one, at 16.7. The number of major organal systems in living things also comes in at around seven.

The present biogeographic problem provides another setting for exploring the applicability of the first Attribute model; i.e., might the seven-regions faunal model be represented as a dendrogram of relations consistent with a “lazy universe” interpretation of net macroevolutionary results? To find out, the seven-regions presence/absence data were reset into proportional (and other) formulations on the basis of the lengthy list of measures set out in [41], a well-known review. In all, thirty-six measures were investigated (about one quarter of the coefficients in the Hayek list could not be adapted to cluster analysis), with the results arranged according to Hayek’s three-category division of association measure types (similarity coefficients, matching coefficients, and traditional association measures). For each of the thirty-six measures, four clustering models were applied: flexible linkage, complete linkage, unweighted pair-group average, and weighted pair-group average. This resulted in 144 analyses in all. Single linkage clustering was not investigated because of the well-known chaining issue (which makes it an inferior choice, on cleanly separated spherical data, to other hierarchical clustering algorithms such as the complete and average linkage models).

The results obtained must be weighed against three important prior considerations. First, and even granting a “natural system” interpretation of mammal faunal patterns, the combined phylogenetic and spatial arrangement model applied here is likely not more than an approximation to a hypothetically “optimum” joint solution. Second, the Holt et al. arrangement yielded regions that, unlike relations among sets of individual taxa at a species or genus level, were nearly maximally distinct from one another, an initial situation never ideal for clustering operations. Third, with so many kinds of association measures organizing the scores input to the clustering algorithms, it was expected that only some relatively small proportion, if any, of the 144 clusterings would fit the 7-4-3-2-1 “five order” (per Figure 3) maximum-likelihood tree pattern.

In the end, only eight of the thirty-six measures examined produced at least some trees that exactly fit one of the two seven-tipped maximum-likelihood tree (“MLT”) models. Six of the eight were complete linkage models falling within the similarity coefficients group; the remaining two, from measures in the matching coefficients group, produced two successes each from the flexible linkage and weighted pair-group average models. In all, therefore, 10 of the 144 trees fit the model. These lukewarm results, even granting the “prior considerations” mentioned above, are tempered somewhat by the fact that another 34 of the 144 analyses produced trees that narrowly missed the MLT standard; these recorded a bifurcation at the third hierarchical order level (see Figure 3 above) instead of the second; these are results that might be reversed with only very slight changes to the prior phylogenetic or regional associations models. Further, (1) all ten of the “successes” exhibited the same MLT form, the type not illustrated in Figure 3, and (2) of the total of forty-four successes/near successes, all but five displayed the same arrangement of regional tips, as shown (for all ten of the “successes”) in Figure 4.

It should also be pointed out that there are dozens of possible five-order, seven-tipped tree hierarchy forms, and that the particular Marine–Australian–Madagascan–Latin American–Afro-Tethyan–Southeast Asian order of tip assignment here is but one of ninety-six possible such permutations (only ninety-six, because tips two and three, and five six and seven, are structurally interchangeable).

A further set of cluster analyses was run on permutations of the raw presence-absence data for the 140 mammal families across the 7 regions as a check; the analyses were stratified by (1) simple presence-absence data, and the same, weighted by cosmopolitanism values (2) initial matrices formulations consisting of Pearson correlation coefficients, and Euclidean distances, and (3) the four clustering models listed earlier. In all, thirty-two analyses were thus performed. Of these, three returned the results shown in Figure 4, while there were two additional “near misses”.

Further interpretation of this pattern of results is beyond our scope here, but the relatively sizeable number of Figure 4-like outcomes overall is worthy of further consideration.

## 5. Conclusions

On the basis of the data provided by Holt et al. [13] and the studies presented here, it seems reasonable to conclude that a seven-region model (including a marine realm) most efficiently depicts the worldwide pattern of mammal family dispersion and diversification. This is true whether one chooses to view the results as a possible example of a “natural law-based” pattern, or merely an efficient phenetic device. Regarding the “natural law” interpretation, the present data are neither conclusively in favor, nor dismissive.

Note, however, that it is significant that if there were a “natural law” interpretation ever established, it would be possible to use this to aid in resolving purely classificatory issues at the family level. Specifically, final arguments regarding lumping and splitting efforts based on the cladistic reconstructions could be rooted in part on the observed spatial patterns, and also to what degree of completeness (variation explained approaching one hundred percent) a posed revision is self-specifying as described here and in CHS’s 1983 papers. To be meaningful, of course, the phylogenetic status of all the taxon units would also have to have been firmly established, but the same spatial diversification tool might also be applied to help clarify this by identifying which posed relations most efficiently harmonize the spatial and phylogenetic models.

## Figures and Tables

**Table 1:** The 7 × 7 matrix of mammal families faunal relations scores among the regions depicted in Figure 1. Each cell in Table 1 contains four values: the top left value represents similarities scores (after [1]), the top right value is the number of “forms common to”, the bottom left value is the zeroth moment “common-tos”, and the bottom right value is the negative zeroth moment “common-tos.” See Table S1 in the Supplementary Materials for its construction.

	Holarctic	Latin American	Afro-Tethyan	Madagascan	Southeast Asian	Marine	Australian
Holarctic	47, 47 142, 187	−36, 22 81, 73	−21, 27 106, 83	−46, 6 32, 10	−24, 26 98, 84	−43, 6 23, 19	−49, 8 42, 14
Latin American	−36, 22 81, 73	55, 55 121, 264	−68, 14 66, 32	−60, 4 23, 5	−68, 14 60, 38	−51, 6 23, 19	−63, 6 33, 9
Afro-Tethyan	−21, 27 106, 83	−68, 14 66, 32	55, 55 156, 229	−39, 11 47, 30	−11, 33 118, 113	−54, 5 23, 12	−48, 11 54, 23
Madagascan	−46, 6 32, 10	−60, 4 23,5	−39, 11 47, 30	17, 17 53, 66	−45, 9 43, 20	−31, 0 0, 0	−25, 6 33, 9
Southeast Asian	−24, 26 98, 84	−68, 14 60, 38	−11, 33 118, 113	−45, 9 43, 20	55, 55 157, 228	−60, 3 11, 10	−30, 17 60, 59
Marine (Oceanic)	−43, 6 23, 19	−51, 6 23, 19	−54, 5 23, 12	−31, 0 0, 0	−60, 3 11, 10	14, 14 37, 61	−31, 3 14, 7
Australian	−49, 8 42, 14	−63, 6 33, 9	−48, 11 54, 23	−25, 6 33, 9	−30, 17 60, 59	−31, 3 14, 7	26, 26 77, 105

**Table 2:** Ten sets of descriptive statistics across the five classification models.

	No. 1	No. 2	No. 3	No. 4	No. 5	No. 6	No. 7	No. 8	No. 9	No. 10
5-region model	45.000	95.000	0.7308	2.230	1.607	−33.000	19.000	51.720	12.500	40.900
6-region model	43.333	111.000	0.7450	2.596	1.857	−31.166	18.500	60.890	13.533	50.867
7-region model	38.429	106.143	0.6518	2.805	1.921	−31.367	15.163	55.570	11.286	47.143
8-region model	36.625	115.875	0.6542	3.225	2.093	−29.797	14.484	62.049	11.321	54.357
9-region model	34.111	118.111	0.6253	3.535	2.193	−28.852	13.124	62.828	10.500	55.917

**Table 3:** Statistics pertaining to the efficiency of the seven-region model here (see text for discussion).

Statistic Description	5-Region Model (5 Regressions)	6-Region Model (6 Regressions)	7-Region Model (7 Regressions)	8-Region Model (8 Regressions)	9-Region Model (9 Regressions)
Mean overall signifs. for r regressions	0.0803	0.0046	0.00047	0.00025	0.000097
Mean of r overall *r*^2^’s in model	0.9943	0.9969	0.9964	0.9893	0.9828
S.d. of r overall *r*^2^’s in model	0.0068	0.0020	0.0025	0.0044	0.0070
Mean, 2 lowest (of the *r*) f stats.	47.7	127.8	157.9	78.4	59.2
Mean, 3 lowest (of the *r*) f stats.	98.6	139.3	171.5	96.1	68.3
Mean, 4 lowest (of the *r*) f stats.	131.8	234.8	253.7	112.9	73.2
Mean, of all but the highest (of the *r*) f stats.	131.8	316.0	426.6	131.6	100.8
Mean, of all but the 2 highest (of the *r*) f stats.	98.6	234.8	309.1	126.2	93.2

## Data Availability

Data available in the Supplementary Materials.

## Supplementary Materials

The following supporting information can be downloaded at https://www.mdpi.com/article/10.3390/life13040873/s1, Supplementary Materials_short.xlsx.
